# Factors influencing carrion communities are only partially consistent with those of deadwood necromass

**DOI:** 10.1007/s00442-023-05327-8

**Published:** 2023-01-25

**Authors:** Christian von Hoermann, M. Eric Benbow, Ann-Marie Rottler-Hoermann, Tomáš Lackner, David Sommer, Joseph P. Receveur, Claus Bässler, Marco Heurich, Jörg Müller

**Affiliations:** 1 Department of Conservation and Research, Bavarian Forest National Park, Freyunger Str. 2, 94481 Grafenau, Germany; 2 Department of Entomology, Department of Osteopathic Specialties, AgBioResearch and Ecology, Evolution and Behavior Program, Michigan State University, East Lansing, MI 48824 USA; 3 Institute of Evolutionary Ecology and Conservation Genomics, University of Ulm, Albert-Einstein Allee 11, 89069 Ulm, Germany; 4 Department of Zoology, Faculty of Science, Charles University, Vinicna 7, 12844 Prague, Czech Republic; 5 Department of Ecology, Faculty of Environmental Sciences, Czech University of Life Sciences in Prague, Kamycka 1176, 16521 Praha, Czech Republic; 6 Faculty of Biological Sciences, Institute for Ecology, Evolution and Diversity, Conservation Biology, Goethe-University Frankfurt, 60438 Frankfurt am Main, Germany; 7 Bavarian Forest National Park, Freyunger Str. 2, 94481 Grafenau, Germany; 8 Department of Visitor Management and National Park Monitoring, Bavarian Forest National Park, 94481 Grafenau, Germany; 9 Chair of Wildlife Ecology and Wildlife Management, University of Freiburg, 79106 Freiburg, Germany; 10 Institute for Forest and Wildlife Management, Inland Norway University of Applied Science, 2480 Koppang, Norway; 11 Field Station Fabrikschleichach, Department of Animal Ecology and Tropical Biology, University of Würzburg, 96181 Rauhenebrach, Germany

**Keywords:** Carrion, Multi-taxa communities, Decay stage, Microbes, Necrophilous beetles

## Abstract

Research on decomposer communities has traditionally focused on plant litter or deadwood. Even though carrion forms highly nutrient-rich necromass that enhance ecosystem heterogeneity, the factors influencing saprophytic communities remain largely unknown. For deadwood, experiments have shown that different drivers determine beetles (i.e., decay stage, microclimate, and space), fungi (i.e., decay stage and tree species) and bacteria (decay stage only) assemblages. To test the hypothesis that similar factors also structure carrion communities, we sampled 29 carcasses exposed for 30 days that included *Cervus elaphus* (*N* = 6), *Capreolus capreolus* (*N* = 18), and *Vulpes vulpes* (*N* = 5) in a mountain forest throughout decomposition. Beetles were collected with pitfall traps, while microbial communities were characterized using amplicon sequencing. Assemblages were determined with a focus from rare to dominant species using Hill numbers. With increasing focus on dominant species, the relative importance of carcass identity on beetles and space on bacteria increased, while only succession and microclimate remained relevant for fungi. For beetle and bacteria with focus on dominant species, host identity was more important than microclimate, which is in marked contrast to deadwood. We conclude that factors influencing carrion saprophytic assemblages show some consistency, but also differences from those of deadwood assemblages, suggesting that short-lived carrion and long-lasting deadwood both provide a resource pulse with different adaptions in insects and microbes. As with deadwood, a high diversity of carcass species under multiple decay stages and different microclimates support a diverse decomposer community.

**Supplementary Information:**

The online version contains supplementary material available at 10.1007/s00442-023-05327-8.

## Introduction

Dead organic matter of any type like wood, leaves, dung, and animal carrion—called necromass—forms an essential resource for a broad range of saprophytic organisms (Moore et al. 2004; Benbow et al. 2019). Diverse communities of microbes (e.g., bacteria and fungi), invertebrates (e.g., insects and arachnids), as well as vertebrate scavengers (e.g., vultures, coyotes) depend on this decaying organic matter (Catts and Goff 1992; DeVault et al. 2003; Benbow et al. 2015; Metcalf et al. 2016). Major ecological functions of the necrobiome are digestion, fragmentation/penetration, nutrient and microbe transport and dispersal, detoxification, and predation (Benbow et al. 2019). Necromass decomposition driven by community members of the necrobiome contributes to nutrient recycling, energy flow, and the limitation of biomass accumulation as important ecosystem processes (Benbow et al. 2019).

Research on necrobiome communities has traditionally focused on decaying plant matter such as litter or deadwood (Swift et al. 1979). Decomposition of leaf litter returns more than 50% of net primary production to soil in terrestrial ecosystems (Wardle et al. 2004). In addition to litter, deadwood is an important necromass resource, promoting diversity of saproxylic (deadwood dependent) invertebrate species (Stokland et al. 2012; Seibold et al. 2016, 2021; Ulyshen and Šobotník 2018). Deadwood comprises all non-living woody biomass and does not include leaf litter. Deadwood can be either lying on the ground, standing, or in the soil (FAO 2004) and can be classified in the following three main components: logs or lying deadwood, dead-standing trees or snags, and stumps (Paletto and Tosi 2010; Ligot et al. 2012). Abiotic and biotic factors such as decay stage, climate, substrate quality, host species, or spatial distance, that determine the composition of the deadwood and leaf litter necrobiome are well understood (García‐Palacios et al. 2013; Lee et al. 2014; Purahong et al. 2016; Baldrian 2017; Krah et al. 2018; Müller et al. 2020), whereas relatively little is known for carrion necrobiome communities.

As a nutrient-rich resource with high turnover rates, carrion forms an important resource pulse in many ecosystems (Bump et al. 2009), and therefore provides an essential food source for a vast diversity of microbes, invertebrates, and vertebrates (Benbow et al. 2015). However, research on animal-derived necromass has so far mainly focused on decomposition rates, and potential use of necrophagous insects in forensics (e.g., Campobasso et al. 2001; Simmons et al. 2010; Haelewaters et al. 2015; Zeariya et al. 2015; Tembe and Makuratirwa 2021), rather than illuminating the complex ecology and network interactions of its decomposer communities.

Regarding factors influencing communities associated with carrion necromass, Farwig et al. (2014) showed that carrion decomposition rate depended on the composition, but not taxa abundances, of insect scavenger assemblages. In carrion-associated insects, the change in composition (e.g., relative beetle or fly abundances to community assemblages) depicted a loss of large dominant species at higher ambient temperatures (Farwig et al. 2014). Pechal et al. (2013) summarized several factors influencing microbial community assembly and function during vertebrate carrion decomposition, and others reported tissue type differences (Dickson et al. 2011), temperature effects (Ward et al. 1998; Carter and Tibbett 2006; Barton et al. 2013), and influences of soil pH (Haslam and Tibbett 2009). However, only limited studies were conducted to explain how variation in the species of the once living vertebrates and the local environment affects the colonization of microbes (Crippen et al. 2015).

Newsome et al. (2021) recently suggested a multi-taxa analysis of microbe, insect, and vertebrate taxa to determine ecological indicators that reflect consequences of carrion persistence and decomposition rates along with parasite presence, vegetation, and soil assessments. Thus, carrion monitoring might become an essential tool for examining key ecological processes similar to those established for plant-derived substrates (e.g., Keuskamp et al. 2013). Bridging plant-derived and carrion necromass research will improve our understanding of the ecology of decaying organic matter inhabiting organisms and their contribution to whole-ecosystem functioning (Benbow et al. 2019). In the long term, comparative work can provide useful information for sustainable forest management and ecosystem restoration, as targeted manipulation of animal and plant necromass can return key processes critical to nutrient cycling and decomposition (Benbow et al. 2019).

To expand ecological understanding of the major determinants of carrion necromass communities and processing, we tested the hypothesis that communities of beetles, bacteria, and fungi were structured by similar drivers as those of deadwood. Across two deadwood experiments, Müller et al. (2020) found highly consistent factors influencing beetle, fungi, and bacterial communities: decay stage and tree species were most important for deadwood fungi and decay stage was most important for structuring deadwood beetle and bacteria communities. In saproxylic beetles, microclimate was the driving indicator for community structure compared to host identity (Müller et al. 2020). To examine, whether these findings in deadwood communities can be transferred to the carrion necrobiome, we exposed 29 wildlife carcasses of three mammal species in a low range mountain forest. We then sampled bacteria, fungi, and necrophilous beetle communities over time for 6 months. Specifically, we predicted, based on the observations in deadwood, that carrion beetle assemblages would be determined by decomposition stage (p1), microclimate (p2), and distance among carcasses (p3); fungi assemblages by decomposition stage (p4) and carrion species type (p5) and bacteria only by decomposition stage (p6).

## Materials and methods

### Study area and design

The study was conducted in the Bavarian Forest National Park (BFNP) in southeastern Germany. Mixed mountain forests mostly cover the 98% forested area and consist of a majority of Norway spruce (*Picea abies*), European beech (*Fagus sylvatica*), and Silver fir (*Abies alba*). The region has a cold and humid continental climate with some maritime influence from the west. The large mammal species pool included the herbivores, roe deer (*Capreolus capreolus*) and red deer (*Cervus elaphus*); the carnivores, Eurasian lynx (*Lynx lynx*), gray wolf (*Canis lupus*), red fox (*Vulpes vulpes*), and pine marten (*Martes martes*); and the omnivore wild boar (*Sus scrofa*). From June to November 2018, in multiple trials [see exposition dates for each individual carcass (designated by Plot-ID) in the Online Resource 1 Table S1], we exposed 18 roe deer, six red deer, and five red fox carcasses (all defrosted for representing the fresh stage of decomposition at the time of exposure) aboveground on 21 plots where the following were recorded: individual carcass fresh weights, exposition type (fixed locations versus random sites), plot coordinates, elevation, and exposition date (Online Resource 1 Table S1). Red fox and roe deer carcasses were obtained from roadkills in the BFNP. Red deer carcasses were obtained from roadkills and from wildlife management (culled by professional hunters) in the BFNP. All 29 exposed wildlife carcasses were declared by the BFNP wildlife management as animal remains not suitable for human consumption. Because of carcass supply from unpredictable roadkill situations and cullings in the BFNP, unequal carcass numbers per carrion type (18 roe deer, six red deer, and five red foxes) were exposed and sampled during our experimental period. A single carcass was placed on each of 21 plots and allowed to decompose for 30 days. For a more detailed schedule and description of the wildlife carcass exposition, see the Online Resource 1 (Table S1) and von Hoermann et al. (2021). All 21 plots were arranged under semi-open canopies of mixed montane forests. The distances between carcasses ranged from 883 m to 35 km. Carcasses at random sites were randomly spaced in the environment (minimum distance of 883 m) to avoid microbial cross-contamination. At fixed locations with the same coordinate, a new carcass was exposed no earlier than 2 months after the previous carcass exposition (see ‘Exposition date’ in the Online Resource 1 Table S1) to diminish cross-contamination effects.

### Biodiversity sampling

Necrophilous insects were sampled using pitfall traps 2 days after the carcasses were exposed. A total of 7 trap-emptying events per carcass were conducted over the whole decomposition period at 2, 4, 6, 9, 16, 23, and 30 days after day 0 of exposure (von Hoermann et al. 2018, 2020). All distinct stages of decomposition namely, fresh, bloated, post-bloating, advanced decay, and dry remains, based on large-scale succession data (Matuszewski et al. 2010, 2011; von Hoermann et al. 2018, 2020) were covered by these seven sampling events. The five distinct and morphologically described stages of decomposition, identified via forensic, and carrion ecological studies (Benbow et al. 2015), are compiled and described in our previous work (Table 1 in von Hoermann et al. 2012) and were modified after Powers (2005), Centeno et al. (2002), Goff (2009), and Anderson and Vanlaerhoven (1996). Two pitfall traps were installed directly at each carcass. One trap was mounted adjacent to the carcass mouth, and the other adjacent to the anus, to provide two locations for sampling carrion insects attracted to the carcass (Dekeirsschieter et al. 2011). For a more detailed pitfall trapping description, see von Hoermann et al. (2021). To provide a consistent sample time period for each trapping event, pitfall trap lids were removed 48 h before content collection. Consequently, each insect sampling event lasted 48 h. At each trap-emptying event, each carcass was photographed for later morphological evaluation and classification of decomposition stages (von Hoermann et al. 2012). All insects were preserved in 70% ethanol. Beetles from groups Silphidae, Staphylinidae, Scarabaeidae, Histeridae, Dermestidae, and ‘Coleoptera rest’ (all other beetle taxonomic groups) were identified to species by external specialists from bureaus, Bavarian State Collection for Zoology in Munich, Czech University of Life Sciences in Prague and Charles University in Prague and stored at the BFNP. 79 obligatory and facultative necrophilous species for beetles formed the basis for subsequent statistical analyses.

Carrion-inhabiting microbial communities were sampled by swabbing the oral mucosa of carcasses at 0, 2, 4, 6, 16, 23, and 30 days after day 0 of exposure. Not enough amounts of DNA for getting reliable bacterial sequencing results were recorded for 12 sampled carcasses. Consequently, a subset of 17 carcasses (10 roe deer, 2 red deer, and 5 red foxes) was used for later analyses. Microbial communities were sampled by three ‘in and out’-movements with a sterile cotton applicator of the upper palate of the mouth followed by the same procedure under the tongue. Directly after sample collection, each cotton swab (Copan FLOQSwabs^™^, MAST Diagnostica GmbH, Reinfeld, Germany) with adherent carrion material was stored in 600 µl RNA*later*^®^ stabilizing and protecting medium with immediate RNase inactivation (RNA*later*^®^, Merck, Darmstadt, Germany) for later analysis in the laboratory. Total genomic DNA was isolated with a PowerMag Soil DNA extraction kit (Qiagen, addition of 15 mg/ml lysozyme during lysis). Two gene regions were selected for sequencing to obtain both bacterial and fungal communities. Both regions have been previously successful in characterizing the microbial species (bacteria, archaea, and fungi) in a controlled, laboratory-based terrestrial vertebrate carrion decomposition study (Metcalf et al. 2016).

Bacterial community composition was determined by high-throughput sequencing of the 16S rRNA gene (V4 region). PCR amplification was performed using the primers (515f and 806r) and a dual-indexing strategy previously described by Caporaso et al. (2012) and Kozich et al. (2013). From the same carrion oral mucosa swabs, fungi were sampled on a subset of 20 carcasses (13 roe deer, 3 red deer, and 4 red foxes) following the same sampling and storing procedure as described for bacteria, again by high-throughput sequencing based here on amplification of the fungal internal transcribed spacer (ITS) region. Not enough amounts of DNA for getting reliable fungal sequencing results were recorded for nine sampled carcasses. Consequently, a subset of 20 carcasses was used for later analyses. The fungal primers, gITS7 and ITS4 (Ihrmark et al. 2012), with Fluidigm indexing oligonucleotides (CS1/CS2) added, were amplified in a primary PCR reaction, followed by cleanup with a QIAQuick PCR Purification kit (Qiagen). Resulting products were normalized and pooled by the MSU RTSF Genomics Core ( https://rtsf.natsci.msu.edu/genomics/, Michigan State University, USA) before sequencing on an Illumina MiSeq platform (2 × 250 bp reads). Initial filtering and demultiplexing was performed using DADA2 implemented in QIIME 2 (Callahan et al. 2016; Bolyen et al. 2019). Bacterial Amplicon Sequencing Variants (ASVs) were taxonomically assigned using a naïve Bayes classifier and the SILVA reference database (v 13.2, 99%, Quast et al. 2012; Bokulich et al. 2018). Fungal ITS sequence data were subjected to similar processing and demultiplexing protocols within QIIME 2 as done for bacteria, except the UNITE reference (alpha version 8.2 UNITE 99% similarity reference set) was used to facilitate taxonomic assignment for fungal ITS sequence reads (Bates et al. 2013; Nilsson et al. 2019; Abarenkov et al. 2020). Sequencing data for this project have been deposited in the NCBI Sequence Read Archive (SRA) under the project PRJNA796973.

### Microclimate conditions

Local, on-site temperature was recorded every 30 min during the 30 day decomposition period using data loggers (Thermochron iButton, Whitewater, WI, USA) mounted on a wooden stick in a standardized height of 1 m above each exposed carcass. At this height above the ground, flying carrion insects are commonly foraging for food in the local area of a carrion resource (von Hoermann, personal observation). This setting was shown to provide a reliable on-site measuring during 1 month for the abiotic parameter temperature as an important predictor for the abundance and diversity of carrion-associated beetles (von Hoermann et al. 2018, 2020). Furthermore, the setting was already successfully applied for describing bacterial community dynamics during in situ carcass decomposition (Pascual et al. 2017).

### Data analysis

All analyses were conducted in R (www.r-project.org). To discriminate roles of biotic and abiotic factors on local necrobiome communities associated with the exposed carcasses, we calculated distance/dissimilarity matrices of the species composition, carrion species type, environmental [abiotic factors microclimate (local, on-site temperature) and spatial distance] and decay stage (days 0, 2, 4, 6, 9, 16, 23, and 30 after day 0 of carcass exposure) data based on a framework of Hill numbers (or the effective species number, Hill 1973). Hill (1973) unified different diversity indices into Hill numbers, applying an increasing weighting from rare (Hill number of diversity order *q* = 0) to dominant (Hill number of diversity order *q* = 2) species. For using Hill numbers to quantify species diversity, see Ellison (2010) and Müller et al. (2020). The Hill number of diversity order *q* = 0 reduces to species richness in which species abundance is ignored. Order *q* = 1 reduces to Shannon diversity (effective number of common species), and *q* = 2 to Simpson diversity (effective number of dominant species). We used Sørensen-type measures to quantify the compositional dissimilarity between necrophilous species communities across sampling events (successional days 0, 2, 4, 6, 9, 16, 23, and 30 after day 0 of carrion exposition) and carrion species types (Chao et al. 2014). Defining *q* = 0 in the class of Sørensen-type measures results in the richness-based Sørensen index (equal weighting of all species for focusing on individuals of rare species). The equal weighting of all individuals was achieved by setting *q* = 1 (abundance-based Horn index; focusing on common species). Defining *q* = 2 resulted in the abundance-based Morisita–Horn index (focusing on dominant species in the assemblages with low weighting of rare species) (Chiu et al. 2014).

Spatial distance (based on Gauss–Krüger coordinates of each carcass) was extracted in all analyses using the Euclidean distance measure to compute the distances between the plot coordinates of each carcass sampling event (distance matrix computation). For carrion species type (roe deer versus red deer versus red fox), a categorical variable called ‘carrion type’ was used. The distance of succession stage was extracted from the numerical days of sampling (days 0, 2, 4, 6, 9, 16, 23, and 30) and the corresponding variable was designated and used as ‘decay stage’ in the rest of the manuscript. We used fine-scaled numerical days of sampling instead of a rather coarse resolution of specific and separated stages of decomposition. For instance, during the cold season, one of our exposed fox carcasses was in the morphologically described ‘fresh stage’ up to 6 days *post-mortem* (days 0, 2, 4, and 6). Numerical days of sampling automatically imply the physicochemical mediated sequence of decay stages for differently sized carrion types with different decomposition rates during different climatic conditions. Microclimate was extracted from a numerical variable representing the computed mean values throughout the 30-min temperature-recording intervals within 48 h of trap-opening.

The distance matrices for microclimate, decay stage, carrion type, and spatial distance were all standardized and then subjected to variance partitioning using function *varpart* in the package vegan based on the adjusted *R*^2^, taking into account the number of terms in the model (Borcard et al. 1992).

## Results

From the 29 exposed carcasses, 12,500 specimens representing 79 necrophilous beetle species were collected. The amplicon sequencing on the subset of 17 and 20 carcasses produced 1820 bacteria and 3726 fungal ASVs, respectively (for more details, refer to NCBI SRA under the project PRJNA796973; e.g., https://trace.ncbi.nlm.nih.gov/Traces/sra/?run=SRR17608725&krona=on).

Variation partitioning showed decreasing *R*^2^ values from necrophilous beetles, to fungi and to bacterial assemblages. Compared to beetles, the model predictability in microbes was very low.

**Fig. 1:**
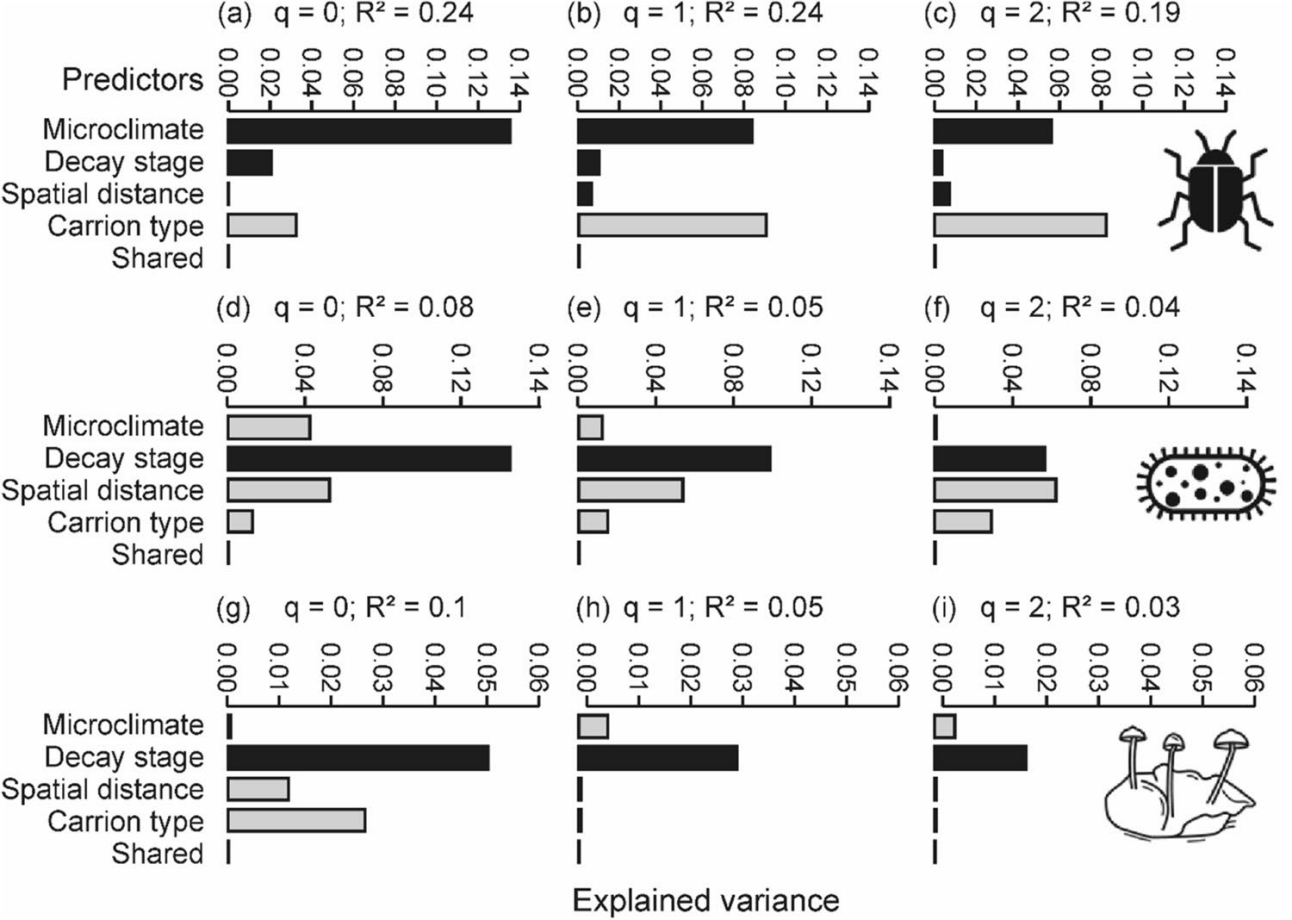
Unique and shared explained variances for necrophilous beetles, bacteria, and fungi assemblages. Variances were determined in a distance-based redundancy analysis using the Sørensen (*q* = 0, focusing on rare species), Horn (*q* = 1, focusing on common species), and Morisita–Horn (*q* = 2, focusing on dominant species) indexes in an increasing weighting of abundant species. Predictors with the same tendencies along the Hill-series as in deadwood necromass (see Müller et al. 2020) are marked in black. The *R*^2^ are adjusted values. Note that the x-axis for beetles, bacteria, and fungi are in different scales

Microclimate was the most important driver of beetle assemblage composition when rare necrophilous species were weighted higher (*q* = 0), followed by carrion type, decay stage, and spatial distance (Fig. 1 a). With increasing weighting of abundant beetle species by increasing q, carrion type increased considerably in importance reaching the first rank for common species (*q* = 1), and for dominant species (*q* = 2) (Fig. 1 b, c). Assemblages of bacteria showed carrion decay stage as the main predictor for rare species and for common species followed by spatial distance, microclimate, and carrion type (Fig. 1 d, e). With increasing q, the importance of decay stage for bacterial assemblage composition decreased and was exceeded by spatial distance for dominant species (Fig. 1 f). Like in necrophilous beetle assemblages, the importance of carrion type exceeded that of microclimate for common and dominant species (Fig. 1e, f). Decay stage was the most important driver of fungi assemblage composition for all three orders (Online Resource 1 Fig. S1), followed by carrion type, spatial distance, and microclimate when rare carrion-associated species were higher weighted (*q* = 0, Fig. 1g). Unlike in necrophilous beetle and bacteria assemblages, the importance of carrion type exceeded that of microclimate for rare species (Fig. 1 g). Spatial distance was of much higher importance for rare fungal species (*q* = 0, Fig. 1 g) compared to common and dominant species (*q* = 1 & 2, Fig. 1h, i).

Spatial distance was more important for bacteria and rare fungi species than beetles. For the bacterial community composition, we observed a consistent contribution of spatial distance over all values of q (for rare, common, and dominant species; Fig. 1d, e, f). Decay stage was more important for the microbiome than necrophilous beetles. Carrion type was of particular importance for beetle and rare fungi assemblages. The shared explained variance of the predictor set was low for both necrophilous beetles and the carrion microbiome (bacteria and fungi), underlining the independence of the predictor set (Fig. 1a–i).

## Discussion

The goal of this study was to evaluate and model the relative effects of biotic (carrion type and decay stage) and abiotic factors (microclimate and spatial distance) on beetle, bacteria, and fungi communities known to recycle carrion. Overall, our results revealed that several drivers affected this necrobiome community, but that specific factors differentially affected each community. Microclimate was particularly important for necrophilous beetle community composition (p2), but was exceeded by carrion type for common and dominant beetle assemblages. Contrary to saproxylic beetles, decomposition stage and space were only of minor importance (refuting p1, p3). Decay stage and spatial distance were the main drivers of bacterial community composition (refuting p6 as the solely determinant), whereas decay stage (p4), carrion type (p5), and spatial distance dictated the community composition of rare fungi species.

### The importance of microclimate and carrion type on necrophilous beetle community composition

From rare to dominant species, we found microclimate (p2) to be an important driver of necrophilous beetle assemblages, similar to that reported for deadwood, with a similar decreasing trend from rare to dominant beetle species were observed (Müller et al. 2020). Experiments in deadwood research manipulating microclimate conditions revealed significantly different xylophagous and saproxylic beetle communities (Vodka et al. 2009; Bouget et al. 2011; Seibold et al. 2018). For communities of carrion scavengers, it is known that variation in environmental conditions changes the species composition of groups, such as vertebrates, flies, or dung beetles (DeVault et al. 2004; Fiedler et al. 2008; Rosenlew and Roslin 2008). For instance, poikilothermic insects require warmer temperatures for an optimal metabolism and other physiological processes (Colinet et al. 2015). Accordingly, Baz et al. (2007) showed a decreasing species richness of insect scavenger assemblages with decreasing temperature. With regard to temperature, von Hoermann et al. (2018, 2020) found higher abundances of the dominant silphid and copronecrophagous dung beetle communities at piglet carcasses with higher ambient temperatures. Dawson et al. (2021) showed an increasing invertebrate species richness with ambient temperature using pig carcasses and human bodies. They detected seasonal differences in beetle and ant species richness, and found that ambient temperature rather than decomposition progression influenced the species richness of carrion flies (Dawson et al. 2021). In our study, decomposition progression (driver decay stage) was also of minor importance for beetle community composition (refuting p1) and is in contrast to results for beetles in deadwood (Müller et al. 2020). In this case, the slow decomposition of deadwood seems to provide more distinct niches over time than that of fast carrion decomposition. Based on our results, we confirm the conclusion of Dawson et al. (2021) that it is important to consider ambient temperature when analyzing insect community composition on the carrion resource.

With increasing weighting of dominant beetle species, microclimate was exceeded by the second most important driver, carrion type. Therefore, carrion type and not decomposition progression (refuting p1) was the second important driver of necrophilous beetle community composition. The relation of carrion type to microclimate in carrion beetle communities stands in contrast to deadwood saproxylic beetle communities, where microclimate was consistently the primary driver compared to the host species (Müller et al. 2020).

In the carrion system, the effect of carrion species size on common and dominant beetle assemblages can be recognized in the carcass-size-dependent occurrence of the dominant carrion beetle *Necrodes littoralis* (Coleoptera: Silphidae). This sixth most frequent species in our samples is known to be highly abundant and to breed primarily at large carcasses such as red deer by consuming decaying tissues and preying on larvae of blowflies (Frątczak and Matuszewski 2014; Bajerlein et al. 2018). Also, *Creophilus maxillosus* (Coleoptera: Staphylinidae), the tenth most frequent species in our pitfall traps, is a common visitor on different sized vertebrate carcasses ranging from 5 to 70 kg (Matuszewski et al. 2016) and breeds at larger resources. During a study exposing small vertebrate carrion (stillborn piglet carcasses with an average weight of 1.4 kg), Weithmann et al. (2021) reported the complete absence of this predatory species, suggesting that small piglet cadavers are not the appropriate resource for the large rove beetle *C. maxillosus*. Thus, our results underline the importance of different carrion species and sizes on necrophilous beetle community composition. However, to differentiate carrion species from the effect of size (weight) will require future research.

### The importance of decay stage and spatial distance on bacteria community assemblages

As with deadwood (Hu et al. 2017; Tláskal et al. 2017; Müller et al. 2020), we found that decay stage was of high importance for structuring bacterial assemblages, even if exceeded by spatial distance for dominant species (see paragraph after next). We assume that marked physicochemical changes in pH, moisture/humidity, and oxygen over the course of vertebrate tissue decomposition drastically alter the overall bacterial community composition, even outcompeting the ambient microclimate and the influence of the exposed carrion type. Our results support conclusions of Paczkowski and Schütz (2011) who discuss how metabolic by-product production is related to carrion microbial community composition and change during the course of vertebrate tissue decomposition. Skin rupture and leakage of carrion fluids after bloating of the carcass leads to increased oxygen in a microaerophilic or anaerobic condition, favoring aerobic microbial taxa and suppressing obligate anaerobes, accompanied by secondary volatile products (Paczkowski and Schütz 2011). Correspondingly, Pascual et al. (2017) showed a progressive change in bacterial community composition of individual piglet cadavers with time of decomposition.

We found that microclimate was less important to affecting carrion bacteria (and fungi) communities, with the largest effect was found for rare bacteria species. Consequently, microclimate had a minor influence on the composition of dominant bacteria species. Temperature is known to strongly affect microbial species composition through modified optimal growth rates, especially for psychrotrophic or mesophilic species (Ercolini et al. 2009; Paczkowski and Schütz 2011). Benbow et al. (2015) stated that temperature thresholds may be known for some individual microbes but are not well defined for entire communities of microbes. Under natural conditions, the effects of the environment on microbial communities have not been extensively investigated in the carrion system (Benbow et al. 2015). Our study is the first, to the best of our knowledge, to reveal microclimate as a minor determinant of bacterial community composition during carrion decomposition. In both types of necromass (carrion as well as deadwood), community assemblages of bacteria were more strongly determined by succession of decay stages then by microclimate or host species (for deadwood, see Müller et al. 2020).

Contrary to our prediction 6, decay stage was not the only important driver for carrion bacteria, with spatial distance being more important for dominant bacteria species. This finding confirms the prediction by Benbow et al. (2019) that two (or more) patches of deadwood or carrion at different decomposition stages or spatial locations—as was the case with carcass exposition in our study—will support a greater community diversity than two of the same resource patches at the same location or at similar decomposition stages. In contrast to our findings, deadwood microbial communities have not been reported to vary with spatial distance (Müller et al. 2020). Komonen and Müller (2018) showed that most deadwood organisms investigated within a 20 km range revealed a higher dispersal ability than commonly assumed. We suppose that different carrion decomposition stages at different locations (supported by the high influence of decay stage on carrion-associated microbial communities) in combination with distinct local environmental variables (Glynou et al. 2016; Wei et al. 2018) had a strong influence on the microbial community composition for carrion necromass, explaining the low predictability in microbes (see appropriate *R*^2^ values in Fig. 1d–i). The contrasting results of spatial distance for deadwood and carrion microbial communities may be due to known intense microbial interactions with flies that often overwhelm and consume the carrion resource in a way that mixes the exogenous and endogenous microbial communities (Tomberlin et al. 2017; Weatherbee et al. 2017). The significant alteration of bacteria communities by actively dispersing adult blowflies (Pechal 2012) acting as vectors for microbes should explain the importance of spatial distance for carrion microbial assemblages. Community analysis from a broad sampling of blowflies and their larval substrates as suggested by Tomberlin et al. (2017) should be performed in future studies for substantiating our assumption.

### The importance of decay stage on carrion fungi community composition

As for deadwood fungi communities (Müller et al. 2020), we found that carrion decay stage was important for fungal assemblages (confirming p4). We assume that the carrion resource changing nutrient conditions during the course of decomposition affects the overall fungal community composition. This may be explained by the known importance in ammonia and postputrefaction fungi over carrion decomposition (Carter and Tibbett 2003). Ammonia fungi form fruiting structures on soils treated with nitrogenous compounds that release ammonia upon decay (Sagara 1975; termed postputrefaction fungi under natural occurrence, Sagara 1995). Postputrefaction fungi have been reported in association with vertebrate carcasses, such as crows (Fukiharu et al. 2000), rabbits (Takayama and Sagara 1981), snakes (Hilton 1978), and kangaroos (Miller and Hilton 1986). The succession was classified in early and late stages (Sagara 1975). Carter and Tibbett (2003) concluded that the succession of nitrogen utilization and fruiting in ammonia and postputrefaction fungi even provides the basis for a post-burial interval estimation in forensic investigations, substantiating the importance of biotic and chemical changes during the decomposition progression in fungal community assemblages and their succession, as we revealed in our study. Furthermore, Metcalf et al. (2016) found a suite of bacterial and fungal groups that contributed to nitrogen cycling during mammalian carrion decomposition and a reproducible decomposer network that emerged in a predictable temporal period. Fungi in the groups Eurotiales and Ascomycota were strong drivers of community structure in that study (Metcalf et al. 2016). In our results, the Ascomycota was the second most abundant phylum after Basidiomycota (relative abundances larger than one percent) with a noticeable fluctuation during the successive decay stages (Online Resource 1 Fig. S1). Metcalf et al. (2016) confirmed microbial succession during decomposition regardless of soil types, host species, and seasons as a predictable measure of the post-mortem interval, and their results corroborate the ones presented here that carrion decay stage was important for both bacterial and fungal communities compared to microclimate and carrion type. Consequently, carrion type was not as important for the fungi community assemblage as formulated in prediction 5 and as reported for deadwood fungi (Müller et al. 2020). In both types of necromass, species turnover on degrading substrates within hours, days or weeks (for carrion), or the first years (for deadwood) supports the strong effect of decay stage of microbial (bacteria and fungi) assemblages, albeit in exceedingly different periods.

## Conclusions

Our study demonstrates that overall biotic and abiotic drivers affect necrophilous community assembly of carrion necromass at a landscape scale and highlights the importance of carrion type and spatial distance in maintaining a highly diverse necrobiome. Our results promote a comparative necromass research identifying that short-lived carrion and long-lasting deadwood both provide a resource pulse with different conditions and resources for a diverse community of insects, bacteria, and fungi. Understanding such differences in deadwood and carrion necrobiomes may provide new information on how, when, and where these saprophytic organisms contribute to whole-ecosystem functioning in complex landscapes. We emphasize the importance of continuous inputs of undisturbed, aboveground carrion of different species and at different spatial scales to maintain diverse multi-taxa communities associated with recycling carrion necromass and redistributing energy and nutrients in forest ecosystems. Both aspects would be realized in nature by itself, provided that human activities do not quickly remove wildlife carrion.

## Supplementary Information

Below is the link to the electronic supplementary material.

Supplementary file1 (PDF 225 KB)

## Data Availability

Sequencing data for this project have been deposited in the NCBI Sequence Read Archive (SRA) under Project No. PRJNA796973. All relevant data supporting the analyses of necrophilous beetle communities are available from the corresponding author on reasonable request.
