# Supplementary material for: Factors influencing carrion communities are only partially consistent with those of deadwood necromass
Source: Oecologia. 2023 Jan 25;201(2):537–47. doi: 10.1007/s00442-023-05327-8 (PMC9943954; doi:10.1007/s00442-023-05327-8)
Supplement: Supplementary file 1 — Supplementary file1 (PDF 225 KB) [file 442_2023_5327_MOESM1_ESM.pdf]

Electronic Supplementary Material

Manuscript title: Factors influencing carrion communities are only partially consistent with those of deadwood necromass

Authors: Christian von Hoermann*, M. Eric Benbow, Ann-Marie Rottler-Hoermann, Tomáš Lackner, David Sommer, Joseph P. Receveur, Claus Bässler, Marco Heurich, Jörg Müller

* Correspondence: Christian.vonHoermann@npv-bw.bayern.de; Tel.: +49 8552 9600-156

Table S1. Carrion exposition scheme and site attributes in the Bavarian Forest National Park during June – November in 2018 (modified after von Hoermann et al. 2021).

Plot-ID	Exposition type	Carrion species	Fresh weight [kg]	Longitude	Latitude	Exposition date [day of the year]	Elevation [masl]
R-01-I	fixed location	roe deer	19,1	13,419776	48,9467	156	806
R-02-E	random place	roe deer	25,35	13,393355	48,9491	156	878
R-03-I	fixed location	roe deer	14,45	13,202491	49,0995	156	775
R-04-E	random place	roe deer	17,9	13,21265	49,1146	156	808
R-05-I	fixed location	roe deer	17,35	13,524246	48,8822	184	851
R-06-E	random place	roe deer	22,75	13,48961	48,9271	184	1081
R-07-I	fixed location	roe deer	13,45	13,340252	49,0272	184	825
R-08-E	random place	red deer	87,5	13,375025	49,0396	184	1058
F-13-E	random place	fox	4,85	13,414011	48,9379	219	783
F-14-E	random place	fox	6,4	13,194835	49,112	219	774
R-09-I	fixed location	roe deer	14,4	13,41972	48,9467	219	805
R-10-E	random place	roe deer	24,4	13,399378	48,9564	219	933
R-11-I	fixed location	roe deer	20	13,202491	49,0995	219	775
R-12-E	random place	roe deer	21	13,224653	49,1136	219	880
R-15-I	fixed location	roe deer	21	13,524246	48,8822	247	851
R-16-E	random place	roe deer	13,5	13,474573	48,9225	247	1003
R-17-I	fixed location	roe deer	13,5	13,340252	49,0272	247	825
R-18-E	random place	roe deer	24	13,359511	49,0284	247	1071
F-23-E	random place	fox	4,7	13,396476	48,9351	275	843
F-24-E	random place	fox	6	13,226218	49,091	275	656
R-19-I	fixed location	red deer	70	13,419721	48,9467	275	806
R-20-E	random place	red deer	110	13,336821	48,9288	275	791
R-21-I	fixed location	roe deer	24	13,202601	49,0996	275	776
R-22-E	random place	roe deer	25	13,222612	49,0763	275	643
F-29-E	random place	fox	7	13,452245	48,9341	310	820
R-25-I	fixed location	red deer	30	13,524246	48,8822	310	851
R-26-E	random place	roe deer	18	13,46863	48,9367	310	975
R-27-I	fixed location	red deer	90	13,340252	49,0272	310	825
R-28-E	random place	red deer	55	13,336547	49,0349	310	825

In Plot-ID: R stands for deer (roe or red deer), F stands for fox carrion, I designates an intensive carrion placement type (fixed location), E designates an extensive carrion placement type (random place); Fresh weight = unfrozen weight at the time of exposition on day zero; masl = meters above sea level.

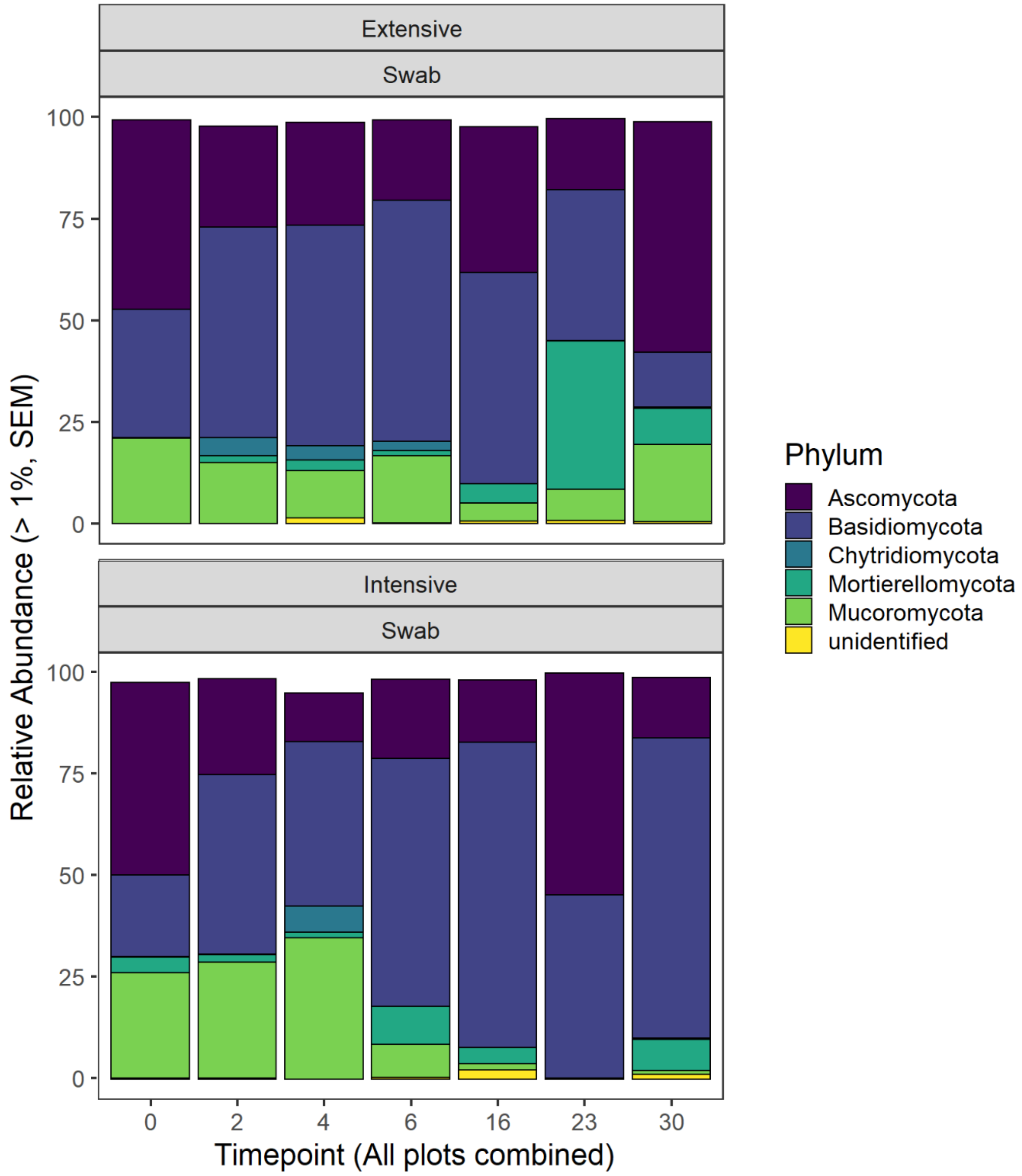

Figure S1. Fluctuation of relative abundances of fungal phyla during decomposition. Successive decay stages (Timepoint) are shown at the x-axis of the taxa plot. Random carrion exposition plots are labeled as ‘Extensive’. Fixed carrion exposition plots are labeled as ‘Intensive’.

References

von Hoermann C, Lackner T, Sommer D, et al. (2021) Carcasses at fixed locations host a higher diversity of necrophilous beetles. Insects 12: 412
